# A Treatise on Medical Study, or on the Method of Studying and of Teaching Medicine

**Published:** 1840-04

**Authors:** 


### ART. VI.

*Traité des Etudes Médicales, ou de la Manière d'étudier et d'enseigner la Médecine.* Par E. FRED. DUBOIS (d'Amiens), Professeur agrégé à la Faculté de Medecine de Paris, &c. &c.—*Bruxelles*, 1838. 12mo, pp. 460.

*A Treatise on Medical Study, or on the Method of Studying and of Teaching Medicine.* By E. FRED. DUBOIS (of Amiens.)

IT can scarcely, we think, be doubted that important changes will take place, within the next few years, in the character of our Medical Faculties, and in the qualifications which will be required from those who seek to enter the ranks of our profession. We feel equally confident that, in whatever changes are effected, *one additional* demand will be made—*a sound preliminary education.* Our reason for assured belief on this point is the universal feeling of its necessity among those bodies who have publicly expressed their opinions by petitions or resolutions. Whatever differences there may be in the plan of the building which is to be erected, all are agreed that it should have this deep and solid foundation. We can scarcely, then, invite the attention of our readers to any topic of more immediate importance than the question—What constitutes a sound preliminary education for him who intends entering upon medical study? This question we propose now to discuss. We shall have to revert, in so doing, to the fundamental principles of all education; and to consider the utility of various branches of knowledge to the growing mind. In taking such a survey, it may appear as if we were quitting our own sphere, and entering upon a range of topics which we have no right to treat of. But nothing can be really foreign to our province which concerns the right training of those to whom our profession is to look for the future maintenance of its dignity and the elevation of its scientific character. Moreover, the *principles* which should guide the purely medical instructor are, or ought to be, derived from those on which education should be conducted from earliest youth. As to readiness for acquiring new information, even the seniors amongst us need aspire after no higher title than that of "children of a larger growth;" and, whilst the varying character of the mind at different epochs is kept in view, the fundamental *principles of education* must be the same for the child and the gray-haired sage, for the man of letters and the physical philosopher.

We believe it to be a most important principle in physiology, which has hitherto received but little attention, that the lower the degree of organization, the more is the character of the being susceptible of influence from external sources. The simpler plants and animals have been found to produce germs capable of being developed into a great variety of forms, according to the conditions under which they live and grow; so great a variety, that naturalists have created, not only species and genera, but families and orders, among beings which really sprang from a common origin. The resemblance between the embryos of higher beings and the permanent forms of the lower is now universally acknowledged; and the resemblance is in no respect more striking than this. It is during the *early* period of the development of the organism that external influences have the greatest effect upon its form and character; and changes may be induced in it at that time, which, at a later period, it would either resist, or entirely succumb to. We shall take another opportunity of enforcing this principle, as one of great importance in physiology. At present we merely bring it forward in illustration of the principle with which we shall start, as lying at the foundation of the educational training of the mind—that it is at the period of the *first development* of each faculty, that it may be most advantageously encouraged, guided, or repressed, according to the strength and tendency which it then manifests.

This principle is by no means new. It was enforced by Bacon in his noble treatise on the Advancement of Learning, to which we shall frequently refer in the present article. "As the wronging or cherishing of seeds or young plants," he remarks, "is that that is most important to their thriving; so the culture or manurance of minds in youth hath such a forcible, though unseen, operation, as hardly any length of time or contention of labour can countervail it afterwards." He was fully alive to the influence of different studies upon the mind; and their consequent value as means of cultivating faculties which might be deficient, or repressing tendencies of injurious strength. "There is no defect in the faculties intellectual but seemeth to have a proper cure contained in some studies; as, for example, if a child be bird-witted, that is, hath not the faculty of attention, the mathematics giveth a remedy thereto; for in them, if the wit be caught away but a moment, one is new to begin; and as sciences have a propriety towards faculties for cure and help, so faculties or powers have a sympathy towards sciences, for excellency or speedy profiting; and therefore it is an enquiry of great wisdom what kinds of wits or natures are most proper for what sciences."

Before entering upon this last question, however, we must pause to enquire what are the real objects of education. We shall confine ourselves at present to the cultivation of the *intellect*;—not overlooking the relative bearing of various branches of knowledge upon the moral feelings, but considering this influence as subordinate to other modes of training more peculiarly applicable to them. That no amount of intellectual attainments can afford a guarantee for the moral rectitude of their possessor, we have a melancholy proof in the profound philosopher from whose writings we have just quoted. But in the choice of the means of intellectual education, we may justly give a preference to those which exercise a beneficial influence on the moral sentiments, over those which have no such tendency. A comprehensive view of the objects of education ought to include the consideration of the best mode of preparing the mind, not only for active participation in the duties assigned to man, in his present state of being, but for entrance on a nobler and purer existence. But as, by the wise ordination of the Creator, the greatest intellectual satisfaction and moral happiness result from that course which is most fitted to discipline or educate man for eternity, the two objects may be regarded as coincident; and, for obvious reasons, we shall direct our present attention to the former only.

In the education of the intellectual powers, then, two chief ends are to be kept in view: *first*, the most advantageous development of these powers themselves; and *second*, the communication of the greatest amount of knowledge capable of being brought into useful application.

Neither of these should be adopted to the exclusion of the other. The system of education still pursued in many of our public seminaries professedly aims at the first only. In some, the study of the classics is relied on as the sole means of accomplishing it; and youths are brought up to make extempore Latin verses, and compose with fluency in Greek, with a profound ignorance of every other branch of knowledge, excepting such as may have been fortunately instilled into them at an earlier period. Against such a system we need scarcely contend; since the class of readers we are addressing must have long since renounced it. Even the addition of pure mathematics does not, we think, furnish a sufficient corrective of its injurious effects. That this study is most valuable, when judiciously conducted, as a means of intellectual discipline, we most readily admit; and we shall presently advert more in detail to the influence it is capable of exerting. But if the pupil is left entirely ignorant, as he too often is, of its *practical* applications, its utility is grievously restricted. A tendency is now gaining ground among a large class of intelligent persons in this country, to regard as the *principal* that which we consider the *subordinate* object of early education. It is thought that the communication of practical knowledge may be itself the instrument, and a sufficient one, of intellectual cultivation. But we are not prepared to go this length; although, as we shall presently show, such a course of instruction may be made to exercise almost all the intellectual faculties in an advantageous manner.

Education may be regarded as either general or special. By *special* education we of course mean the preparation of the mind for some particular employments, which are to be its chief occupation for the remainder of life. Now in *this* department, the acquisition of the greatest amount of knowledge, and of the power of practically applying it, is manifestly the chief end. No one would set a young man to the practice of medicine or law, however well developed might be his intellectual powers, or however large an amount of general knowledge he might have attained, without instructing him in the particular branch which he is to pursue. But it is evident that these instructions will be more efficient, in proportion to the *previous* cultivation of the intellectual faculties, and the *previous* amount of well-digested knowledge, on the foundation of which the newly-communicated ideas may be orderly arranged and firmly upreared. Our opinion, then, is, that *general* education ought to be directed especially to the *first* of the objects we have named; and *special* education to the *second;* but in neither case exclusively. We shall endeavour to show that general education may be so directed, as not only to prepare the intellect for the most advantageous employment of its powers, in the department which may be subsequently chosen for its exercise, but to store up an amount of practical information, which will be useful in almost every subsequent pursuit. It will be obvious that, of two systems equally efficacious for the first object, *that* will be preferable which goes furthest towards the second. And, if our principles be correct, the preliminary or general education will be nearly the same, whatever is to be the ultimate destination of the individual.

We shall now briefly consider some of the branches of knowledge usually included in the early training of the young, in their relations to the development of the intellectual powers, and to their own prospective utility. In doing so, we shall derive many excellent hints from the volume whose title we have placed at the head of our article. With a good deal of French diffuseness of style, and a little of French dogmatism of opinion, this work has much of that sound practical character which marks the other productions of M. Dubois. It is evidently the result of much thought; and coming, as it does, from one who holds an elevated position in the Parisian School of Medicine, we recommend it to the attention of all those who are interesting themselves in the subject of medical education in this country. With his leading principle, that all education ought to proceed from what is general to what is special, we fully coincide. It is the course pointed out to us by many natural analogies; and it is that which is in soundest harmony with the varying powers and wants of man at different periods of his existence. We shall now trace the application of this principle.

The mind of the infant, on its entrance into the world, has been compared by some to a sheet of white paper, on which the educator may impress anything he pleases. We by no means coincide with this doctrine; since it supposes that there is no variety of mental *capability*, at this period, amongst different individuals; but, still, there is much truth in it. We are convinced that the intellectual education of the infant should begin with its moral training; and that both these are closely connected, in the early years of infancy, with attention to its physical development. Nothing, for example, is more prejudicial to the future temper of the child than the habit, so common amongst indulgent mothers, of putting it to the breast as often as it cries. The habit of indulgence once commenced is seldom or never discontinued; and the evil extends itself into every part of the character. But such a matter is rather foreign to our present purpose; and we have referred to it only in illustration of our principle, that the influence exercised on the powers and tendencies of the mind, at the period of their first development, is more important than any which can be put in operation at a subsequent period. Now, in directing our attention to intellectual education, we notice that the first-developed powers of the infant's mind are those connected with *observation.* And the rationale of this is obvious. The intellect at that period may be compared to a seed. It is in a state of dormant capability. It possesses faculties which need to be called into activity; and as warmth, moisture, and oxygen arouse the energies of the germ, and cause it to exert those powers with which it is endowed, so as to develope itself into its perfect form, so do external objects operate on the mind. If all inlets to sensation were closed, we can scarcely imagine that any use could be made of its faculties. It would remain like the seed buried deeply beneath the ground. But as soon as it receives impressions from external objects, and ideas are excited within it, a series of changes or operations commences, which can only terminate with the loss of its powers.

The cultivation of the observing faculties, then, is the first branch of education in the order of time; and it is that which has the most general relation with all others. In no pursuit, no situation in life, will the habit of acute and correct observation be valueless; and we are persuaded that to the medical student much subsequent labour would be saved, if he were earlier taught to have not only *his eyes* but *his ears about him.* The habit can scarcely be commenced too early. "An infant, intently gazing upon an object, or examining it with its little hands and lips, is as usefully employed in the cultivation of intellect as the fondest parent can wish. In the early periods of mental culture more is, however, to be done, in this connexion, by allowing a child full scope for its own efforts, than by any direct exertions which can be made by others. When its attention is fixed upon any object, let it remain so; if possible, let the objects of sense be brought into view under different aspects, and exposed to the examination of different senses. Before words become to a child the signs of voluntary action, all that can be done is to expose it to sensations, and to allow them to fix the attention; but afterwards, more direct efforts may be made, and the attention may be fixed by various other means, beside the mere action of the sensations themselves. ........ It is a most erroneous idea in education that nothing is done except when children are engaged in the usual rudiments of instruction. A child watching the motions of objects around, observing their figure and sounds, examining their structure, is employed in a work which it should be our aim, as much as possible, to aid and encourage, and from which we may expect very valuable results, both on the faculties and furniture of the mind."* In the essay from which we have been quoting will be found many valuable remarks on the proper discipline of the observing faculties, and on the distinction between quickness of sensation and quickness of perception. Those who possess acute sensibility are often deficient in that power of attention which is required for correct observation; whilst, on the other hand, those whose reflective powers are cultivated too exclusively are often so immersed in abstractions as to give no heed to the impressions of external objects. Each of these tendencies may be detected in early childhood; and the efforts of the instructor should be directed to their due regulation. With regard to the extensive, the universal operation of the habit of observation, we may add the judicious remarks of the same author. "The successful acquisition of every science which depends upon experiment, indeed, the acquisition of knowledge of every kind which depends upon the exercise of the perceptive power, the cultivation of the taste, the common concerns of life, the intercourses of civility, and the efforts of benevolence, require the constant exercise of this habit. Whatever method is found to invigorate and correct the observation should be frequently made use of. Till the understanding has made considerable progress, this should be made a leading object in the intellectual culture; and in every period of it, the habit should be frequently brought into exercise. By a proper cultivation of it, the memory and judgment are directly cultivated; and while it strengthens and rouses the energy of the mind, it furnishes it with some of the most serviceable materials for the understanding and imagination."

Next, in generality of character, to the formation of ideas by the impressions of the objects themselves is the acquisition of them through the medium of *language.* This power it is, therefore, the object of the instructor to convey at as early a period as possible. A clear and correct

* Rees's Cyclopædia; Art., *Intellectual Education.*

understanding of the force of words is of the utmost consequence in every period of the intellectual progress. On the care which is early taken in furthering this object depend, in a great measure, the future development of the understanding and the acquisition of knowledge. The tendency of many systems of education has been to familiarize the pupil rather with words than with things; and whilst we guard against this, by cultivating the observing faculties, not only in childhood, but throughout the period of youth, we should also take especial care that *definite* ideas be attached to the words employed. A simple method of attaining both these purposes, is to require from the pupil minute verbal descriptions of objects to which his attention has been directed; and in so doing, we shall be training him up in a habit which is itself of great utility. Too often, as soon as the study of language is commenced, we see the child's attention withdrawn from the world around him, and directed only to his books; and hence it is that medical students need so much time and training to learn "how to observe." On the other hand, if the study of language be not systematically pursued, we rarely find that definiteness and propriety in the use of words, which are necessary alike for the communication of knowledge to others, and for the reception of it ourselves. A deficiency in this respect is evident in the writings of many self-taught men. Take, for instance, the works of Hunter: and if he, instead of learning from the great book of nature, had devoted himself to the acquisition and condensation of the opinions of others, we venture to say that (until his mind had been disciplined by such a process,) his language would have been even more obscure than it is. Every one knows how many fierce controversies have taken place on abstract subjects, which have depended only on a slight difference in the meaning attached to words; and a mere vagueness of expression will often be a serious obstacle to the advancement of truth, by preventing the secure employment of the fact or opinion stated, as a basis for others. This is particularly the case in those departments of physiology which bear upon metaphysics, as we have formerly taken occasion to show.

M. Dubois lays great stress on this department of preliminary education; and, we think, with justice. There must, he remarks, be a complete harmony between the teacher and his pupils in regard to the use of language. They should be able to follow, without difficulty, all his trains of thought, and to become thorough partakers in his ideas; placing their feet, as it were, in the footsteps of him who is advancing in their front. This they cannot do, if they are incapable of appreciating his instructions, from want of aptitude in comprehending the force of language; and we have known many instances, in which a lecturer, who has succeeded in carrying along with him, through an abstruse discussion, a considerable proportion of his class, has been misunderstood by some, whilst others have given up all idea of following him, in consequence of such a deficiency. That mere plain English will not always convey the speaker's or author's meaning so well as some fanciful almost absurd, expression, newly coined, perhaps, from his own brain, who that has read the Essays of Elia will not acknowledge? For ourselves, we seldom think it accordant with our critical dignity to depart from the forms of language; but the lecturer may often have recourse to an exuberance of illustration, or pointedness of expression, which it might not become the author to adopt. We believe, then, that felicity in the use of language is a habit which may, like acuteness of observation, be most advantageously cultivated in very early life; and that, if so cultivated, the best foundation will be laid for the subsequent discipline of the higher intellectual powers, and for the attainment of knowledge on any subject.

We are disposed to agree with M. Dubois, that the acquisition of a foreign language, of almost any nation, is the best means of making us well acquainted with our own; and, further, that the study of the classics has an advantage, in this respect, over that of any modern tongue. Moreover, to the scientific man, some amount of classical knowledge is almost essential to the object we have just been adverting to—the ready comprehension of the meaning of terms. That the learned in all ages should have agreed to base their nomenclature upon the languages most universally understood, cannot be wondered at; and we do not perceive how the system can be advantageously changed. No one can tell how difficult it is to communicate knowledge on some subjects (e. g. Natural History,) to those who are ignorant of the classics, and consequently of the import of the words derived from them, until he has tried the experiment, as we have. But we have a strong impression that, for those whose taste does not lead them to the study of the higher classic authors, the acquisition of modern languages will serve nearly the same purpose, as a means of intellectual discipline, and will be of far more practical utility.

Now that we are upon this topic, we may briefly state our opinion of the value of classical study, as a branch of general education, and therefore of that training which should precede entrance on medical study, and which should be tested by preliminary examination. We think that no other *single pursuit* can exercise the same beneficial influence on the mind, by cultivating so large a portion of its faculties in an advantageous manner; and that none is so well adapted to *indiscriminate* application in large establishments, from its simultaneous action on many distinct powers and tendencies of the intellect. Command of the attention, ready and capacious memory, exact and delicate discrimination, judgment in the application of principles and in the choice of words, a sort of indescribable aptitude in the conception of things as a whole and in their details, the power of realising abstract notions, a facility in combining simple and in analyzing complex ideas—these are but a few of the objects whose fulfilment may be reasonably anticipated from a well-conducted course of classical study; and a youth thus trained will go forth into the world with many advantages over him who has received only a scientific education, besides those which result from the cultivation of the taste and imagination. But these advantages are rather in the discipline which his mind has received than in the knowledge with which it has been stored; and the youth thus trained must not forget that his education, though of a higher order, has advanced less far in preparing him for the business of the world than if his attention had been, from the first, directed towards objects of more practical utility. If he make good use of his time and opportunities, he will generally make rapid progress in the attainment of the latter, unless the early cultivation of his observing faculties have been much neglected. But he will have to educate himself, as it were, over again. It is not a little remarkable that a large proportion of those who have most distinguished themselves in extending the boundaries of human knowledge, have either been almost self-taught, or, if at school, have been reputed dull boys; and the exceptions have been principally among those who have chanced to be placed under an instructor of more than ordinary enlightenment and sagacity; whilst, on the other hand, it is a common remark, that few of those who distinguish themselves in scholastic exercises are subsequently heard much of by the public. The first-class men and senior wranglers in our universities, after enjoying a short-lived reputation amongst a limited circle, too often feel satisfied with what they have done; and, considering their past acquirements as the end and not the means, allow their mental powers to fall into disuse, at the period of their greatest vigour, or exercise them only upon unprofitable subjects. It is not only in our own country that this may be observed. *Les héros du College*, as M. Dubois calls them, seem to be similarly inert in France; and, in his opinion, as in ours, the system must be faulty which produces such results.

For the reasons we have above given, we fully accord with the practice pursued by most of our medical boards—of obtaining proof of the candidate's knowledge of the Latin language; and we are not disposed to quarrel with the College of Physicians for requiring Greek also. But we think that this knowledge should be tested at a much earlier period than that of the medical examination; and the regulation of the London University appears to us a very judicious one. We have known cases in which individuals have gone through a regular course of classical study, and have derived great benefit from its discipline; and who yet, from natural deficiency of literary taste, and from thorough devotion to the acquirement of professional and scientific knowledge, may have so far forgotten, in a few years, the languages they had acquired, as to need much preparation for undergoing an examination in them. If it be acknowledged that their chief use is the preparation of the intellect for being advantageously employed on other subjects, the proper time for examining into the qualifications of the student in this respect, is when he is just entering upon his new pursuits; not when he has completed his medical curriculum, and been devoting weeks or months to *get up* as much Latin or Greek as will enable him *to pass*, which he forgets again as speedily. We have met with young men who were more afraid of being rejected for their Latin at Apothecaries' Hall (this was before it was made, as now, a separate examination,) than for ignorance of any other department. Is not this a practical satire upon the system? If they were well informed upon professional subjects, what benefit would they derive from the *cramming* process, which enabled them, by means of interlinear translations and other such aids, to master as much of Celsus and Gregory as would enable them to put on a bold front before the worshipful examiners? The object of this and of every other requirement comprised in the preliminary examination of the University of London, we take to be twofold: first, to secure such a *preparedness* of mind, on the part of the student, as may enable him to enter with advantage on medical study; and second, to ensure his being able to mix in the world with such an amount of general knowledge as should be possessed by every man practising a liberal profession. On neither of these grounds do we think the requirements in the least too high; in fact, we should be very glad to see them in some departments a little more stringent.

We shall now return to the point at which we diverged from our regular path to consider the benefits of classical study; and shall enquire into the best means of cultivating those reasoning faculties which so rapidly unfold themselves in childhood, when early preparation has been made for their exercise by the culture of the perceptive powers. The direct training of a judicious instructor we believe to be more effectual in guiding the development of these powers than any course of study can be. The phenomena of the external world may be made the subjects, not only of observation, but of inferential processes; and the habit of employing the reasoning powers upon these can scarcely be too early commenced. At first, the inferences to which the pupil is led, must be of the simplest character ; they may gradually be rendered more complex. Among the methods of cultivating the purely reasoning faculties, none is, perhaps, so available as that which exercises them upon the simple relations of *number*, *time*, and *space*. Although these ideas are for the most part abstract, yet they are so immediately connected with sensorial impressions, that they are in general readily comprehended. When the fundamental notions are once established, the reasoning processes based upon them may be carried to almost any extent. It will be seen that, in placing the acquisition of these ideas next in order to that of words, we are following out the principle we formerly laid down; since they are the *most general* of any of the more precise notions connected with external objects; and the simple reasoning processes, founded upon them, are the best that could be devised for the exercise of the mind in that stage of development.

Many persons feel a difficulty in comparing numerical or mathematical reasoning with that of any other kind whose results are less certain. The difference is simply this. In the former, the premises are so simple and restricted, that the mind, feeling that no disturbing causes *can* affect the conclusion, assents to it with *certainty*. At every step of a calculation or demonstration, (supposing it, of course, to have been correctly performed,) the mind is as well satisfied of truth as at the commencement. The highest knowledge is thus derived, by inferential reasoning, from the axioms and definitions on which the simplest conclusions are at first erected; and the mind, which has pursued the enquiry throughout, and given its full assent at every step, feels as thorough a conviction of the final truth as of the fundamental propositions. In that kind of inferential reasoning, on the other hand, which forms a part of what is called *moral evidence*, the premises are not so restricted, and it seldom happens that the mind can exclude the *possibility* of a contrary result. Each step is, therefore, attended with a greater or less amount of doubt; and when many consecutive inferences are drawn, the probability of error increases in a very rapid ratio. We may illustrate this by the process of the surveyor, in laying down an extensive country. He first measures a base line, which is but short, in comparison with the distances he is afterwards to calculate. He then makes this one side of a triangle, whose angles he measures, and whose other sides he can thus calculate. These sides he makes the bases of other triangles, and carries on this process to any required extent. Now it is curious that, in such a train of reasoning, there should be a mathematical certainty with regard to the reasoning itself, and a moral probability only as to the data upon which it is founded. Any error in the measurement of the base line will affect the whole series of calculations; and, if there be any imperfection in the instrument by which the angles are measured, a new error will be created at each stage, so that the final result will be very wide of the truth. Here, the mind will give full assent to the reasoning itself, provided it can feel assured of the data. It is worth notice, in regard to the perfection to which processes of this kind have been carried, that in some of the extensive government surveys in this country and in India, the *base of verification*, measured at the conclusion, has differed from the calculated distance by not more than a few inches, when the triangulation has been carried over hundreds of miles.

We can scarcely imagine any intellectual discipline more adapted than mathematical study to develope and direct a certain section of the powers of the understanding; but their deficiency lies in their limited scope. "The pure mathematics," says Bacon, most truly, "do remedy and cure many defects in the wit and faculties intellectual. For if the wit be dull, they sharpen it; if too wandering, they fix it; if too inherent in the sense, they abstract it." "If there were nothing valuable in the mathematical sciences," observes another excellent writer, "for the uses of human life, yet they are well worth our study; for by perpetual examples, they teach us to conceive with clearness, to connect our ideas in a train of dependence, to reason with strength and demonstration, and to distinguish between truth and falsehood. Something of these sciences should be studied by every one; and that, as Mr. Locke expresses it, not so much to make us mathematicians as to make us reasonable creatures." But it must not be forgotten that, in the exclusive pursuit of this study, the powers of observation, of discrimination, and judgment, are almost all neglected; so that the mere mathematician comes into the world with a much less advantageous preparation than the mere classic. By being conversant with truths of an obvious and simple character only, "he does not learn that kind of caution and severe examination, which are required in other sciences—for enabling us to judge whether the statements on which we proceed are true, and whether they include the whole truth which ought to enter into the investigation. He thus acquires a habit of too great facility in the admission of data or premises, which is the part of every investigation which the physical or mental enquirer scrutinizes with the most anxious care,—and too great confidence in the mere force of reasoning, without adequate attention to the previous processes of investigation, on which all reasoning must be founded. It has been accordingly remarked, by Mr. Stewart, and other accurate observers of intellectual character, that mere mathematicians are apt to be exceedingly credulous, in regard both to opinions and matters of testimony."*

To remedy what is injurious, and to supply what is deficient in mathematical study, we regard the study of the physical sciences as most admirably adapted. In the more exact among them, such as mechanics and astronomy, the close reasoning of the mathematics is applied to phenomena which are the objects of sense. The perceptive faculties are, therefore, exercised in observing these; the powers of discrimination and

* Abercrombie on the Intellectual Powers. Fifth Edition, p. 259.

comparison are employed in classifying them according to their analogy or dissimilarity; and the habit of generalizing with caution and certainty is acquired. The converse process of deduction cannot be better learned than by tracing the complex influence of the simple principle of gravitation, or rather mutual attraction, among the various bodies which form the solar system.

There is one influence of the study of astronomy, well pointed out by Sir J. Herschel, which is most valuable to all, and to none more so than the medical student. "In entering upon any scientific pursuit, one of the student's first endeavours ought to be to prepare his mind for the reception of truth, by dismissing, or at least loosening his hold on, all such crude and hastily-adopted notions respecting the objects and relations he is about to examine, as may tend to embarrass or mislead him; and to strengthen himself, by something of an effort and a resolve, for the unprejudiced admission of any conclusion which shall appear to be supported by careful observation and logical argument, even should it prove of a nature adverse to notions he may have previously formed for himself, or taken up, without examination, on the credit of others. Such an effort is, in fact, a commencement of that intellectual discipline, which forms one of the most important ends of all science. It is the first movement of approach towards that state of mental purity which alone can fit us for a full and steady perception of moral beauty, as well as physical adaptation. It is the 'euphrasy and rue' with which we must purge our sight before we can receive and contemplate, as they are the lineaments of truth and nature. There is no science which, more than astronomy, stands in need of such a preparation, or draws more largely on that intellectual liberality, which is ready to adopt whatever is demonstrated, or concede whatever is rendered highly probable, however new and uncommon the points of view may be, in which objects the most familiar may thereby become placed. Almost all its conclusions stand in open and striking contradiction with those of superficial and vulgar observation, and, with what appears to every one, until he has understood and weighed the proofs to the contrary, the most positive evidence of the senses."*

We are unable to account for the very general neglect of the physical sciences in early education. To us it seems the most natural thing possible, to show to the pupil the practical applications of the abstract notions of number, space, and time, and of the processes by which he builds upon these, at the very period when he is undergoing this discipline. What, for example, can be simpler than to make a child understand the properties of the lever at the time when he is learning the rule of proportion? Simple sums may, by an easy process, be worked mechanically; and thus the numerical and physical notions will be fixed in the mind together, and will strengthen each other. In the same manner, when the pupil learns to *square* a number, he may be *shown* that similar areas are to each other as the squares of their linear dimensions;†

* Treatise on Astronomy, p. 1.

† A very curious and apposite illustration of the importance of this kind of knowledge to the physiologist may be found in the erroneous statement, which has been copied from one work to another, that the capacity of the arterial tubes increases in proportion and, by a very simple mechanical contrivance, the fact that the intensity of light, attraction, &c., diminish, as the squares of the distances increase, may be rendered obvious to him. In like manner, we would make what practical applications are possible, at every stage of the pupil's progress in more abstract investigations; and, in this way, not only will the intellectual discipline be more complete, but a fund of useful knowledge will be laid up. A young person thus trained will be prepared to enter with advantage on investigations of a much higher and more complex nature, when others must commence a previous discipline, which, at that late period, will be comparatively painful, and perhaps inefficient.

We are very glad to perceive that the principle on which we have thus been dwelling has met with so able and influential an advocate as Professor Daniell. Near the commencement of his very excellent Introduction to the Study of Chemical Philosophy, we find the following remarks. "There are two great mistakes, which are commonly committed by those who enter upon a systematic course of physical enquiry; and not only by those who are commencing, but by those who undertake to direct such studies: the first is, the neglecting to form a proper connexion with previously-acquired knowledge,—the under-valuing the results of their ordinary experience as parts of the system, as the first rounds of the intellectual ladder by which they aspire to scale the loftier heights of philosophy; and the second is, the substitution of names for things,—the vague acquirement of certain terms, certain forms of expression, instead of a real understanding of objects and principles to which they have been applied—'terms of ignorance and of superficial contemplation,' as Lord Bacon calls them. The process may be repulsive to the too-common self-sufficiency of imperfect knowledge; but when invited to reflect and reason upon the simple observations of childhood, (simple indeed, but not more easy than those which he will be called upon progressively to make,) the student ought to feel no more offence than when, in the outset of his geometrical studies, he is referred to the axioms or self-evident truths, that 'things equal to the same are equal to one another,' and 'the whole is greater than its parts.' It is from the known that he must ascend to the unknown; and it is all-important that he make his footing sure, and miss no step by the way."

This last observation we hold to be of great importance. The learner to their distance from the heart; it being supposed that at each bifurcation, the capacity of the branches is greater than that of the parent trunk. This notion is manifestly inconsistent with the facts ascertained by Poisseuille, who has shown that the pressure of the blood against the sides of the vessels is almost exactly the same in every part of the body, instead of diminishing, as it would do, with the increased capacity of the tubes. The cause of the error has been recently explained by Mr. Ferneley, (Med. Gaz., Dec. 7, 1839.) It is true that the sum of the *diameters* of the branches is considerably greater than that of the trunk. Thus a trunk 7 lines across may divide into two branches of 5 lines each, or a trunk of 17 into three branches of 10, 10, and 9½; but when their areas are compared, the correspondence is as close as can be reasonably expected, when the nature of the measurements is taken into account. In the first case, the area of the trunk is represented by the square of 7, that is, 49; whilst the area of each branch will be 25, and the sum of the two will be 50. In the second instance, the area of the trunk will be 17 squared, or 289, whilst that of the branches is the sum of 100, 100, and 90¼, making 290¼. Physiologists are much indebted to Mr. Ferneley for making this correction. Like the problem of Columbus's egg, the thing is simple enough when explained.

should, if possible, have nothing to unlearn. Every one knows how much easier it is for truth to find admittance into a mind unoccupied than into one prejudiced by the residence of error. In our progress through the sciences, therefore, we should pass from what is certain to what is uncertain, from what is simple to what is complex; and we shall find that this is exactly what we do in commencing with what is general, and pursuing our enquiries into what is special. For example, medicine is one of the most special of all the sciences, and at the same time the most uncertain; it concerns only the abnormal or disordered vital actions of one species of living beings. Pathology and physiology are, therefore, but branches of a higher science—that of the vital actions in general. But man is only one out of many hundred thousand species of living beings with which this globe is peopled; and whatever may be the difference in practical value between the physiology and pathology of his system and those of the zoophyte or plant, in a merely scientific point of view they are on a level. Medicine, then, is but a small section of the vast science of biology. Now, although organized beings have actions peculiar to themselves, and governed by their own laws, they are still subject to the more general laws of matter. It may, indeed, be argued, with little possibility of error, that what are called *vital* properties are originally inherent in matter, and only developed or caused to manifest themselves when that matter is, by the process of organization, placed in the circumstances required for their operation; just as iron, when brought within the influence of a magnet, itself shows magnetical properties which previously lay dormant. If this be true, the science of organized matter is itself a section or branch of that of matter in general. We find, on a little consideration, that matter possesses certain universal properties, and others restricted to particular forms of it. Thus the mutual attraction of its masses is never absent; it is common to every form of matter, and operates under all circumstances. It is probable that all matter possesses electrical properties, but these are manifested only under peculiar circumstances; and we do not, therefore, regard it as unphilosophical to suppose that all matter may possess what we term vital properties, which are only manifested under circumstances still more restricted. However this may be, it is certain that living organized beings are subject to *all* the other laws of matter; and that we cannot investigate their strictly *vital* properties with any hope of more than limited success, until we are in a condition to exclude all the operations of more general causes.

Hence, then, it is obviously important, that he who aspires after something more than the power of applying practically the knowledge which others have attained of the structure and actions of the human body, who aims at giving to medicine a more certain character, and thus raising it from the opprobrium in which it lies at present, should prepare himself by a command of those general principles to which the laws of life are but subordinate. The profound remarks of Bacon are applicable to this as to every department of knowledge: "After the distribution of particular arts and sciences, men have abandoned universality or philosophia prima, which cannot but cease and stop all progression; for no perfect discovery can be made upon a flat or level; neither is it possible to discover the more remote and deeper parts of any science, if you stand but upon the level of the same science, and ascend not unto a higher science." Of this *philosophia prima* he elsewhere says, "But because the distributions and partitions of knowledge are not like several lines that meet in one angle, and so touch but in a point, but are like the branches of a tree that meet in a stem, which hath a dimension and quantity of entireness and continuance, before it come to discontinue and break itself into arms and boughs; therefore it is good, before we enter into the former distribution, to erect and constitute one universal science, by the name of philosophia prima, primitive or summary philosophy, as the main or common way, before we come where the ways part and divide themselves."

By this *philosophia prima*, or *scientia scientiarum*, as Bacon elsewhere terms it, we understand those principles which should be our guide in the pursuit of any kind of knowledge that can be brought under the domain of general laws. They depend upon the relation between the mind of man and his position as "minister et interpres naturæ." They are nowhere more clearly set forth than in the admirable "Introduction to the Study of Natural Philosophy," by Sir J. Herschel; a work which we should rejoice to see made a text-book in the higher classes of our schools and colleges. But, alas, the day which shall behold Terence and Aristophanes put aside for Herschel or Somerville is not yet come. When every student of medicine shall be thus prepared, we do indeed anticipate the rapid advance of our science.

These principles of philosophising cannot be taught in any method so advantageous as that which we have already suggested as the best suited to the general wants of the student's mind. With the early foundation we have recommended, he will have little difficulty (always supposing him to have the advantage of an instructor who can properly adapt his particular plan to the mind of his pupil) in making himself acquainted with those general laws of matter, at rest and in motion, which govern the phenomena comprehended in the sciences of statics and dynamics. He will thus have a few comprehensive ideas, which may be pursued into greater or less detail, as his time and inclination permit, in those subordinate branches which are commonly spoken of as mechanics, astronomy, hydrostatics, hydraulics, and pneumatics. In such a course, he would gradually perceive that he was coming into a new domain, and that in passing from solids to liquids, and from liquids to aeriform bodies, the operation of the laws which he at first deemed certain is modified in a way he cannot altogether comprehend; and thus he is led to the conception of new forces resulting from the operation of properties which he has not been hitherto led to perceive. Of these, the most important is heat, which is the chief cause of these differences in form; and he accordingly applies himself to the examination of its properties and laws.

In regard to heat, light, electricity, and magnetism, the opinions of philosophers are at present undergoing a very remarkable transition. These were formerly described as *imponderable* forms of matter; but the advance of knowledge and a sounder mode of philosophising have led many to discard this opinion, which may be looked upon as a remnant of the ancient dogmas; and to attribute the phenomena so designated to properties of matter whose laws are to be investigated like those of any other forces. The knowledge of the true nature of sound, and the almost complete demonstration of the analogous character of light, have mainly contributed to the relinquishment of these "personified abstractions;" and, when they are so disposed of, we hope that the vital principle will go along with them. When the forces which produce the phenomena separately referrible to heat, light, electricity, attraction, &c., have been investigated by the student, he will be in a condition to enter upon the science of chemistry, whose phenomena are due to the combined action of these forces. This is a science which we regard as peculiarly adapted to exercise the intellectual powers. The observing faculties are constantly and actively employed; the mind is habituated to the analysis of complex phenomena, and to the search for the true cause of each change out of many possible ones; and reasoning processes of a high order are concerned in the more abstract departments of the science. Its obvious practical utility causes it, as might be expected, to be one of the most popular branches of knowledge amongst those who derive little intellectual gratification from its pursuit. It is now taught in a large proportion of the well-conducted seminaries, both for male and female education, in Scotland; and it is gradually making its way southwards. We believe that to Dr. D. B. Reid's labours this very useful change is principally due.

We are of opinion that, if the pupil be well grounded in the principles of chemistry, whilst passing through his preliminary course of education, a great deal of time may be spared which is now devoted to this science as a branch of medical education. Having got a general knowledge of the science, he will require nothing further than a special knowledge of those departments of it which bear peculiarly upon medicine. Of these, pharmaceutical chemistry may very properly be included with materia medica; whilst some additional knowledge of organic chemistry may be communicated in the courses of animal and vegetable physiology. The student of forensic medicine ought to be conducted through a regular course of practical analysis, for the detection of poisons; but we are disposed to doubt whether so minute a knowledge on this subject need be diffused among the general body of the profession as some imagine; or whether such investigations may not be better conducted by some person especially skilled in them, such as the lecturer on chemistry or forensic medicine in the nearest medical school. To this question, however, and others strictly connected with medical education, we shall return in our next number.

From chemistry the student may advantageously proceed to mineralogy; on which, however, we would not have him dwell long. He should make himself acquainted with the external characters and chemical composition of the minerals which principally compose the crust of the earth; and he will then be prepared to enter upon the study of geology, some knowledge of which now may be regarded as essential to every professional man who wishes to be regarded as a well-informed and liberally-educated gentleman. Here, too, as in chemistry, the science itself makes abundant demands upon the perceptive and reasoning powers, and advantageously exercises almost all the intellectual faculties. As in astronomy, too, there is in the outset a necessity for the absolute dismissal of prejudice, and a determination to submit the mind wholly to the dominion of truth. There is no science which affords so much scope for ingenious speculation; and none in which new phenomena are so constantly arising to confirm or overthrow the hypotheses which have been invented to account for the facts already known. This exercise of mind is extremely advantageous to every one, and especially so to him who is preparing to enter upon the study of medicine. A facility in framing hypotheses, "if attended with an equal facility in laying them aside when they have served their turn, is one of the most valuable qualities a philosopher can possess; while, on the other hand, a bigoted adherence to them, or indeed to peculiar views of any kind, in opposition to the tenor of facts as they arise, is the bane of all philosophy."*

We do not think that a preliminary education can be regarded as complete, which does not include some knowledge of natural history and general physiology. As means of intellectual discipline, we have a very high opinion of them; and there are probably no branches of knowledge that afford, on a very slight acquaintance, so many sources of rational pleasure and moral improvement. We have known many, who received but a trifling amount of early instruction on these subjects, resort to them in after life as their most refreshing and delightful recreation. It seems to us a neglect of the means of instruction placed by the Creator within our reach, if the young are brought up in ignorance of the teachings of the volume of nature. On the advantages of such preliminary instruction to the medical student, we need scarcely now dwell. Such a general acquaintance as he will thus gain with zoology, is the best foundation he can have for the more extended pursuit of comparative anatomy; and upon the latter all certain physiology must be based. We do not see that any higher knowledge of botany is needed by him than he would gain at the same period, except that merely technical acquaintance with the characters of medicinal plants which he should learn from the course of materia medica. But we are again forestalling our future discussion of these subjects.

It may be thought that the range of study we have thus suggested is by far too extensive for ordinary students. Whether it is so or not, however, will depend mainly upon the method in which they are taught. If a knowledge of *facts* is that which the instructor seeks to communicate, the attention had better be confined within narrower limits; if, on the other hand, he deems his pupil ready for the reception of *principles*, his scope can scarcely be too extensive. It is a remarkable characteristic of the present state of almost all the sciences, that while the facts are being multiplied with unprecedented rapidity, the principles are advancing as rapidly; and this not by the uprearing of showy and unstable hypotheses, but by that sure process of rigid induction which not only lays one course upon another, but binds them all more and more firmly together. The aptitude of the pupil for the reception of

* Herschel's Preliminary Discourse, p. 204.

principles will depend very much upon the early training to which he has been subject. If this have been such as we have suggested, and there be no unusual deficiency of mental power, we feel assured that there are few who are not capable of understanding them, and appreciating their value, if they are put forwards in their most simple form, and illustrated in modes sufficiently simple and various. Even if the student be detained until the age of seventeen or eighteen years, in order to pass through such a course, instead of commencing his medical studies at fifteen or sixteen, we should think the time well bestowed in the greater preparedness of mind which he will possess for entering upon the special study of his profession.

The following extract from a valuable essay to which we have already referred expresses in a striking and concise form the advantages which may be expected from a connected course of study in natural science: "Another source of the utility of the mathematics is their subserviency to natural philosophy. To describe the phenomena of the universe, to investigate their causes, and the connexion of these causes, are the principal objects of this science. To mention these objects is nearly all which is necessary to indicate its valuable effects on the mind. The habits of accurate and persevering observation, of investigation, of abstraction, and of correct reasoning, are more or less produced and cultivated by the study of the philosophy of nature. It furnishes abundant scope for the most sublime speculations, and calls forth the noblest exercises of the imagination, yet restrains the mind within the limits of reality. It carries us beyond the boundaries of sense, and lessens our interest in self by increasing our concern with everything around us. It enlarges the comprehension of the soul; for it offers for contemplation the laws of the universe. It prepares the student for an acquaintance with the human mind; for the strictness with which its investigations are conducted prevents that wildness of theorizing which is the bane of science; and forms the habit of cautiously attending to phenomena, in order to ascertain the general laws which regulate them. It aids the cause of religion; for it accustoms the mind to seek for the causes of observed appearances, and leads it from design and regularity to infer an intelligent First Cause."*

To these remarks we shall add, that such a course of study will tend to cultivate that philosophic spirit which is so peculiarly desirable for the medical practitioner; a spirit which, as Dr. T. Brown justly remarked, "is more valuable than any limited attainments in philosophy, and the cultivation of which, therefore, is the most precious advantage that can be derived from the lessons and studies of many academic years; a spirit which is quick to pursue whatever is within reach of human intellect, but which is not less quick to discern the bounds that limit every human enquiry, and which, therefore, in seeking much, seeks only what man may learn; which knows how to distinguish what is just in itself from what is merely accredited by illustrious names; adopting a truth which no one has sanctioned, and rejecting an error of which all approve, with the same calmness as if no judgment were opposed to its

* Rees's Cyclopædia; Art. Intellectual Education.

own; but which, at the same time, alive with congenial feeling to every intellectual excellence, and candid to the weakness from which no excellence is wholly privileged, can dissent and confute without triumph, as it admires without envy; applauding gladly whatever is worthy of applause in a rival system, and venerating the very genius which it demonstrates to have erred."

The mind which is imbued with the principles of general science has a far greater chance of success in any particular branch than that which has directed all its energies to one restricted department alone. When this preparation has been made, there can be no question that the more closely the attention is bent on any one point, the more probably will that point be gained. But much fruitless labour will often be thrown away, and many errors committed, if this be neglected. "It can hardly be pressed forcibly enough on the attention of the student of nature," observes Sir J. Herschel, "that there is scarcely any natural phenomenon which can be fully and completely explained in all its circumstances, without a union of several, perhaps of all, the sciences." Whatever walk of science the enquirer may pursue, he will find it well to be guided not only by the light of his own intellect, but by the reflection of that light from surrounding objects. The higher we rise in philosophical generalization, the more do the principles of the different sciences become interwoven with each other; so that phenomena which at first sight appear to have nothing in common are found to be intimately connected. The rules of investigation and the general method of conducting it, are, or ought to be, the same in all sciences; and he who has fully entered into them in one department will have but little difficulty in following them through others, especially when he is conducted gradually from the simple to the complex.

There is an intellectual satisfaction in the possession of such a range of acquirements, which strongly contrasts with the spirit which characterizes the mind of the trader in science. The latter views every extension of the boundaries of knowledge with anxiety; since it occasions him fresh labour, or diminishes the value of his former ones. The less his acquirements reward him in and for themselves, the larger remuneration does he crave from others. The irksome, the insignificant in his employment press him to the earth, because he cannot oppose to them that high and cheerful courage which accompanies only a knowledge of the real value of his pursuit, and a stedfast devotion to it. Just as sedulously as he severs his own peculiar science from all others, does the true philosopher strive to extend its dominion and restore its connexion with them. New discoveries in the field of his activity are to him a source of pure and intense gratification. Perhaps they fill a chasm which the growth of his ideas had rendered more wide and unseemly; or they place the last stone, the only one wanting to the completion of the structure of his ideas. But even should they produce an opposite effect,—should a new series of truths, a new aspect of nature, a newly-discovered law, overthrow the whole fabric of his knowledge, *he has always loved truth better than his system*, and gladly will he exchange her old and defective form for a new and fairer one—

Impavidum ferient ruinæ.

It is not among the least of the advantages to be expected from such a course of preparatory study as that which we have been recommending, that he who has been judiciously conducted through it is likely to enter upon the study of medicine with sounder notions regarding the value of knowledge, and upon the practice of his profession with more elevated views in regard to its application. We should hear less of those angry bickerings which arise from mercenary jealousy, and from disappointed ambition,—men would be more ready to act like brethren in one noble community, when the question is not who shall amass the greatest fortune, but who shall contribute most, by the extension of the boundaries of his science or by adding to the knowledge of that which it includes, to the welfare of his kind.

Let us once more turn to the pages of the father of the inductive philosophy, and seek his estimate of knowledge: "But the greatest error of all the rest is the mistaking or misplacing of the last or farthest end of knowledge: for men have entered into a desire of learning and knowledge, sometimes upon a natural curiosity and inquisitive appetite; sometimes to entertain their minds with variety and delight; sometimes for ornament and reputation; and sometimes to enable them to victory of wit and contradiction; and seldom sincerely *to give a true account of their gift of reason to the benefit and use of men:* as if there were sought in knowledge a couch whereon to rest a searching and restless spirit; or a terrace for a wandering and variable mind to walk up and down with a fair prospect; or a tower of state, for a proud mind to raise itself upon; or a fort or commanding ground, for strife and contention; or a shop, for profit or sale; and not a rich storehouse, for the glory of the Creator, and the relief of man's estate. But this is that which will indeed dignify and exalt knowledge, if contemplation and action may be more nearly and straitly conjoined and united together than they have been. Howbeit, I do not mean, when I speak of use and action, that end before mentioned of the applying of knowledge to lucre and profession; for I am not ignorant how much that diverteth and interrupteth the prosecution and advancement of knowledge, like unto the golden ball thrown before Atalanta, which, while she goeth aside and stoopeth to pick up, the race is hindered. Neither is my meaning, as was spoken of Socrates, to call philosophy down from heaven to converse upon the earth; that is, to leave natural philosophy aside, and to apply knowledge only to manners and policy. But as both heaven and earth do conspire and contribute to the use and benefit of man, so the end ought to be, from both philosophies to separate and reject vain speculations, and whatsoever is empty and void, and to preserve and augment whatsoever is solid and fruitful; that knowledge may not be, as a courtezan, for pleasure and vanity only, or, as a bondwoman, to acquire and gain to her master's use; but, as a spouse, for generation, fruit, and comfort."

We fear that the *cui bono?* is a question much oftener asked, particularly in this country, than is consistent with the true dignity or end of knowledge. We seldom hear in Britain of men devoting their whole lives and powers to the improvement of the scientific departments of medicine, content to exchange the worldly reward that they might derive from its practice as an art, for the happiness they derive from the pursuit itself, and from the consciousness of a fame well earned by the success of their labours. We would it were in England as in Germany; and that the place of such a man in society were estimated by his intellectual wealth, and not by the house in which he resides and the dinners which he gives. We are not without hope that such a spirit is extending. We shall augur well for science when it is universal.

It may be urged that the prevalence of such a taste as we have been endeavouring to excite will lead too many to desert the practice of their profession for the pursuit of its theory. We do not think so. It is a beautiful instance of the adaptation between the mental constitution of man and the world in which he is placed, that for every variety of occupation there are found some minds which take pleasure in it. It is not every one whose capabilities encourage the hope of success in the pursuit of the higher departments of knowledge; and yet those who are thus deficient may have a peculiar aptitude for carrying into practical operation the principles which have been attained by others. As long as there are diseases to be cured, will there be found men ready to undertake the employment, as certainly as others who feel a distaste for it. How those who devote their energies to the philosophy of medicine, and have no independent means of support, are to be maintained, is a grave and difficult question. It may be thought that either the state or the profession at large should afford assistance to such as have attained a certain degree of eminence; but whether such assistance should be direct, or whether it should not be afforded by throwing open to general competition those situations which are now more frequently disposed of by private interest, may be fairly doubted. We shall not at present enter upon this discussion; but we may take an opportunity of adverting to it when we enter more fully, as we propose to do in a succeeding article, into the *Connexion of the Medical Sciences.*

When some acquaintance with physical science is possessed by *every* medical practitioner, there will, perhaps, be less difficulty in combining attention to practice with some amount of philosophical enquiry, than at present exists. There is a prepossession in the public mind against those who have a scientific reputation, and in favour of those who are known for nothing but their devotion to practice; even though the unemployed hours of the latter may be spent in an unprofitable manner. It will often happen that the man of scientific education will, from his habits of mind, be able to see at a glance that which the mere practitioner discovers after a long and blundering examination. He will be able to perceive where principles can be applied, and where he must trust to experience alone; and he will more certainly judge of the results which may be expected from treatment, and of what may be more safely left to take its own course. In the analysis of difficult phenomena, the explanation of perplexing symptoms, it is that the scientifically-educated physician has the chief superiority over his more empirical colleague; and the advantage of the early culture of physical science, in cherishing those powers of mind which are required for this purpose, and training them to work in unison, are so obvious that it is strange they have been so long overlooked. When such an education is universal, then we anticipate that medical men will find it more easy to combine the scientific and practical departments of their profession. At present, those who devote themselves to the former must live chiefly upon hope.

But there are, even in this country, not a few, we trust, who find in this pursuit so elevated and never-failing a source of happiness, that they would not relinquish it for any mere worldly good to be anticipated from following a different course. And it is not among these, we venture to pronounce, that those jealousies and disputes prevail, which too frequently meet our view, and which are so disgraceful to the votaries of science. All these disputes may, we think, be traced to that misapprehension of the true ends of knowledge against which we have contended. To the man who loves knowledge for its own sake, and for the sake of its beneficial influence on his race, what does it matter whether he alone has attained the elevation, or whether he shares it with others? The prospect below, around, and above him is the same. He has the same animating satisfaction in the review of difficulties overcome;—the same expansion of feeling as he surveys the extent of the domain beneath his feet;—the same delight in the glimpses he discovers of paths which may conduct him to new and yet more valuable acquirements. It is among those in whom the mere love of fame is the strongest,—who seek most eagerly for the applause, not so much of the master spirits of the age, as of the world at large, and for the substantial advantages which that brings with it,—that we observe the keenest sensitiveness to detraction in regard to the value of their attainments, the greater disappointment if it can be proved that they have been at all anticipated in them. Such a seeker after truth can bear the proximity of no other. He must stand alone. He looks at the pinnacle of knowledge, not as the commanding height from which he may take a wider survey of its glorious domain, but as the pedestal on which he may elevate himself above his fellows, that they may fall down and worship him around its base.

Which of these characters are we most inclined to respect and love?

